# Transparent peer review and open data at *Communications Biology*

**DOI:** 10.1038/s42003-019-0489-0

**Published:** 2019-06-24

**Keywords:** Peer review, Research data

## Abstract

As of January 1st 2019, authors submitting manuscripts to *Communications Biology* can choose to publish the reviewer reports and author replies with their articles. The first articles with associated reviewer reports have now been published, representing an important step in our broader journey toward greater openness.

Earlier this year, we announced the introduction of transparent peer review to *Communications Biology* for all manuscripts submitted as of the first of the year^1^. Transparent peer review at *Communications Biology* works identically to that at *Nature Communications*^2^: authors opt in or out of publishing the reviews and replies to the reviewers at the point of acceptance. Reviewers are notified that by accepting to review the manuscript, they consent to publication of their anonymous comments (or, if they prefer, their signed comments). Although it is still too early to report any statistics, we are encouraged by the uptake so far as well as the positive response of the life sciences community to transparent peer review at *Nature Communications*^3^.

We published our first article^4^ with the full history of reviewer reports and author replies on May 8th, and a second^5^ on May 23rd. Others have been published^6–8^ that contain only partial reviewer report histories because the manuscript came to us after having been reviewed at another journal. If a peer review file is present with an article, you can find it along with any other supplementary information (Fig. 1). In line with recent studies published in *Genome Biology*^9^ and *Nature Communications*^10^, we have not noticed any changes in whether or not referees agree to review or in the quality or speed of the reviews received.

**Fig. 1:**
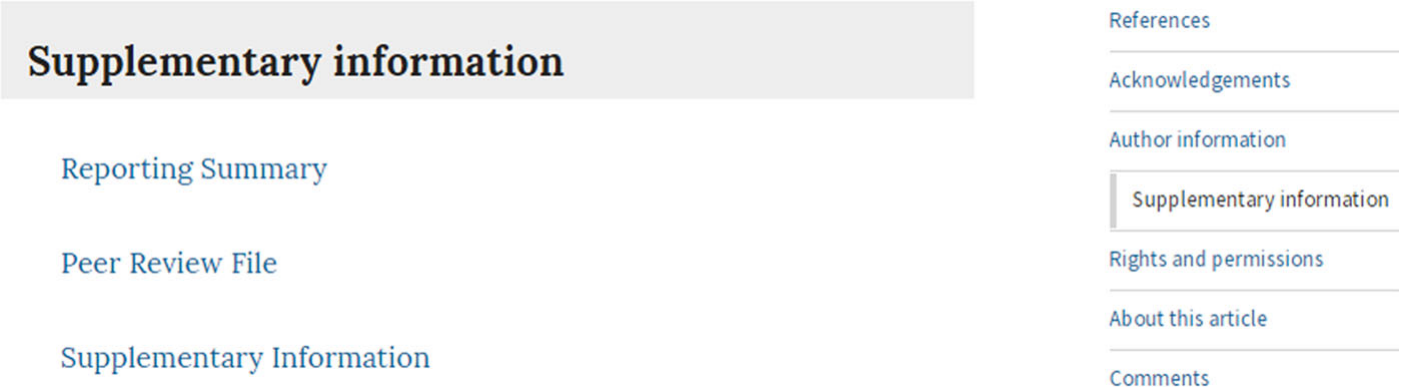
The peer review file is published as supplementary material, together with the Nature Research Reporting Summary and any other supplementary files

So why are we doing this?

We feel strongly that the peer review and publication process at a journal should be as transparent as possible. Providing the peer review file as supplementary information at publication is one part of this. As any experienced editor knows, peer review can be messy, reviewers can disagree, and authors may not be able to fulfill every request of each reviewer. But in the end, this process strengthens the final paper and provides us with the confidence to publish the work in our pages. By opting in to transparent peer review, authors help illuminate a process that is crucial to the communication of rigorous science but has been too long hidden from public view.

By opting in to transparent peer review, authors help illuminate a process that is crucial to the communication of rigorous science but has been too long hidden from public view.

The peer review files published alongside our articles only tell part of the story; they don’t include communication between the editor and authors or the editor and reviewers. They also don’t provide information about why the editors decided to pursue this particular paper in the first place or what their internal discussions may have been. This is for good reason, as these discussions often include confidential information. On the other hand, we aim for more transparency in much of what we do. First and foremost, we do this by maintaining open lines of communication with authors and reviewers so that the reasons behind our editorial decisions are clear. Along with all other Nature Research journals, we also post our median decision times online so that authors can make an informed choice about where to submit their work. And our policies and editorial processes pages contain detailed information about what we do behind the scenes so that readers can be sure that each article published in the journal has gone through a rigorous evaluation process.

Our policies also call for a great deal of transparency from our authors. This includes publication of the Nature Research Reporting Summary, which contains important details about study design, statistical analysis, and accessibility of data, among other things. We also ask authors to give as much detail as possible in their Methods section and enable this by having no word limit for methods.

But we can and should continue to strive for more transparency. One obvious place to improve is in our data availability statements. As with all Nature Research journals, authors of primary research are required to state in the published paper whether and where data are available, and availability is mandated for certain data types. However, a large number of data availability statements continue to simply state that data are available upon request. We can do better. While we acknowledge that not all data types should or reasonably can be made publicly available, we are now asking authors to be more forthcoming in their descriptions of how readers can access the data reported in their papers. At a minimum, information about restrictions on accessibility must be included in the Data Availability Statement. If the data are not made available through a repository, the reasons for this should be made clear.

In addition, we ask that the data underlying plots and graphs in the main figures are available either in the supplementary materials or via an online generalist repository. Since we started requesting these data as a trial earlier this year, over 85% of authors have provided source data with their published paper. Although the source data only include the processed data used to directly plot the graphs presented in the paper, we feel that including these data is important to both reproducibility and transparency, allowing readers to more closely inspect the results than they may have otherwise been able to. This is the same reason we and other Nature Research journals require authors to plot individual points on any bar graphs or convert these graphs to box-plots if the sample size is sufficient (Fig. 2). Given the positive outcome of our trial, we are now making source data mandatory for published papers from today.

**Fig. 2:**
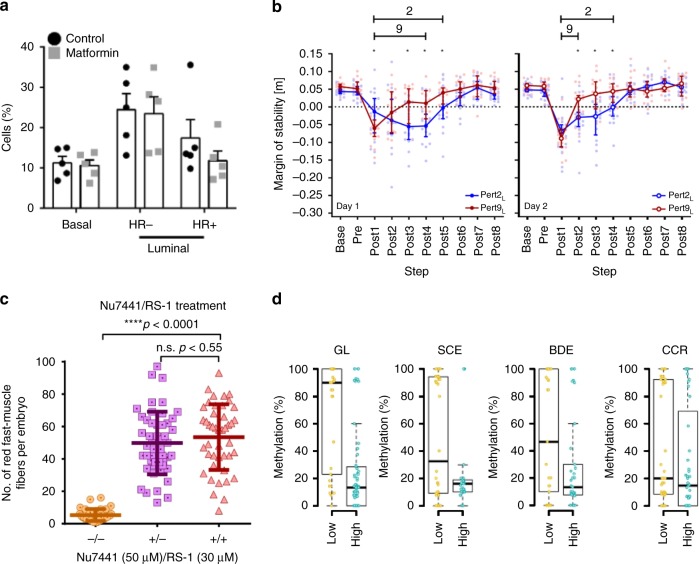
Examples of figures showing data distribution in the plots, instead of bar graphs with mean ± error only. **a** From Fig. 1 of Shehata et al. 2019, doi: 10.1038/s42003-019-0439-x. **b** From Fig. 3 of McCrum et al. 2018, doi: 10.1038/s42003-018-0238-9. **c** From Fig. 2 of Aksoy et al. 2019, doi: 10.1038/s42003-019-0444-0. **d** From Fig. 5 of Fang et al. 2019, doi: 10.1038/s42003-019-0341-6

Transparency in peer review, editorial process, methods reporting, and data availability all contribute to the same goal: increasing trust in science by making sure we publish rigorous, reproducible research.
